# COVID-19 in Autoinflammatory Diseases with Immunosuppressive Treatment

**DOI:** 10.3390/jcm10040605

**Published:** 2021-02-05

**Authors:** Tatjana Welzel, Samuel Dembi Samba, Reinhild Klein, Johannes N. van den Anker, Jasmin B. Kuemmerle-Deschner

**Affiliations:** 1 Pediatric Rheumatology and Autoinflammation Reference Center Tuebingen (arcT), University Children’s Hospital Tuebingen, 72076 Tuebingen, Germany or tatjana.welzel@ukbb.ch (T.W.); Dembi.Samba@med.uni-tuebingen.de (S.D.S.); 2 Pediatric Pharmacology and Pharmacometrics, University Children‘s Hospital Basel (UKBB), University of Basel, 4056 Basel, Switzerland; JohannesN.vandenAnker@ukbb.ch or; 3 Department of Internal Medicine II, Immunopathological Laboratory, 72076 Tuebingen, Germany; Reinhild.Klein@med.uni-tuebingen.de; 4 Divison of Clinical Pharmacology, Children’s National Hospital, 111 Michigan Avenue, NW, Washington, DC 20010, USA

**Keywords:** Interleukin-1, autoinflammatory diseases, CAPS, FMF, coronavirus, SARS-CoV-2 antibody response

## Abstract

COVID-19 disease increases interleukin (IL)-1β release. Anti-IL-1-treatment is effective in IL-1-mediated autoinflammatory diseases (AID). This case series presents COVID-19 in patients with IL-1-mediated and unclassified AID with immunosuppressive therapy (IT). Patient 1 is a 34-year-old woman with an unclassified AID and methotrexate. Patients 2 and 3 (14-year-old girl and 12-year-old boy, respectively) have a Cryopyrin-Associated Periodic Syndrome (*NLRP3 p.Q703K* heterozygous, CAPS) treated with canakinumab 150 mg/month since three and five years, respectively. Patient 4 is a 15-year-old girl who has had familial Mediterranean fever (*MEFV* p.*M694V* homozygous) for 3 years treated with canakinumab 150 mg/month and colchicine. All patients had a mild acute COVID-19 course, particularly the adolescent patients. A few weeks after COVID-19 recovery, both CAPS patients developed increased AID activity, necessitating anti-IL-1-treatment intensification in one patient. At day 100, one out of four patients (25%) showed positive antibody response to SARS-CoV-2. This is one of the first reports providing follow-up data about COVID-19 in AID. The risk for severe acute COVID-19 disease was mild/moderate, but increased AID activity post-COVID-19 was detected. Follow-up data and data combination are needed to expand understanding of COVID-19 and SARS-CoV-2 immunity in AID and the role of IT.

## 1. Introduction

**Figure 1:**
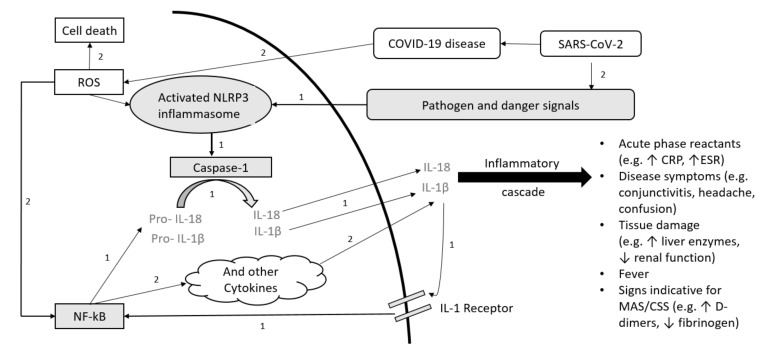
Schematic overview of the pathogenesis for IL-1-mediated autoimmune disease (AID) and COVID-19. (1.) Pathogenesis of NLRP3 Inflammasome associated AID (**gray**): Inflammasome formation is induced by a variety of triggers. Activated NLRP3 subsequently drives caspase-1 activation. Caspase-1 mediates transformation from pro-IL-1β and pro-IL-18 to active IL-1β and IL-18. The positive feedback loop stimulates NF-kB. (2.) SARS-CoV-2 pathogenesis (**white**): SARS-CoV-2 can stimulate a hyperinflammatory immune response with epithelial cell-mediated production of reactive oxygen species (ROS). ROS can stimulate NF-kB and NLRP3. Both pathways (1. and 2.) result in increased cytokine levels with laboratory signs and clinical symptoms associated with hypercytokinemia. Abbreviations: SARS-CoV-2: severe acute respiratory syndrome coronavirus 2; COVID-19: coronavirus disease 2019; ROS: reactive oxygen species; NLRP3: (NOD)-like receptor protein 3; NF-kB: nuclear factor kappa B; IL: interleukin; CRP: C-reactive protein, ESR: Erythrocyte sedimentation rate, MAS: macrophage activation syndrome; CSS: cytokine storm syndrome.

Autoinflammatory diseases (AID) are rare, potentially life-threatening conditions caused by pathogenic gene variants encoding for inflammasomes leading to excessive production of pro-inflammatory cytokines [1]. The NLRP3 inflammasome plays an important role in the pathogenesis of the Cryopyrin-Associated Periodic Syndrome (CAPS) activating caspase-1 (Figure 1). In the familial Mediterranean fever (FMF) pathologic pyrin variants can increase caspase-1 activation. FMF and CAPS are interleukin (IL)-1 mediated AID. IL-1β is one of the most prominent products of inflammasome activation and a key regulator of systemic inflammation (Figure 1); therefore, maintenance anti-IL-1 treatment plays a pivotal role, particularly in IL-1-mediated AID management [2,3,4]. The achievement of low or no disease activity is crucial to avoid morbidity and mortality caused by uncontrolled inflammation. Infections can trigger disease activity in AID. High disease activity results in disease flares with fevers, inflammation of joints, eyes, skin and serous membranes coupled with increased inflammatory markers, and may result in a macrophage activation syndrome, similar to cytokine storm syndrome (CSS) [5,6,7,8,9].

The coronavirus disease 2019 (COVID-19) is associated with cytokine dysregulation, increased IL-1β, tumor necrosis factor (TNF) and IL-6 release with hyperinflammation and risk of CSS (Figure 1) [10,11,12,13,14]. Poor COVID-19 outcome correlates with clinical and laboratory features of the CSS [5]. The avoidance of hypercytokinemia is a pivotal therapeutic aim in COVID-19, similar to AID, and cytokine targeting agents, such as anti-IL-1 treatment, anti-IL-6 treatment or Janus kinase inhibitors may be promising [12,13,15]. Whereas male sex, older age, smoking and comorbidities/underlying diseases, such as cardiovascular and respiratory diseases, diabetes mellitus or obesity seem to be related with higher risk for severe COVID-19 [16,17,18,19], children seem to be less severely affected compared to adults [20,21]. However, in the past months reports about a pediatric inflammatory multisystem syndrome temporally associated with COVID-19 (PIMS-TS)/multisystem inflammatory condition associated with COVID-19 (MIS-C) in children are emerging [22,23,24]. It is known that infection can trigger AID activity. Increased AID activity or AID flares are also associated with cytokine release and hypercytokinemia. Therefore, it might be possible that COVID-19 and the underlying AID may influence each other with increased risk for CSS. Several AID patients have maintenance immunosuppressive treatments (IT), which is also used in COVID-19 treatment. Patients receiving IT in general seem not to be at increased risk to develop COVID-19 or to show more severe disease courses [25,26,27,28]. Up to now, outcome data for patients with underlying AID and confirmed COVID-19 are scarce and particularly follow-up data addressing the underlying AID and its disease activity are missing. Furthermore, no data and follow-up data addressing seroconversion and antibody response to SARS-CoV-2 in AID patients with IT are available.

In this case series, we describe three patients with an IL-1-mediated AID and one patient with an unclassified AID. All patients are treated with maintenance IT and developed COVID-19. We describe: (i) the acute clinical COVID-19 course and furthermore we report follow-up data for the first 100 days after COVID-19 diagnosis addressing, (ii) the clinical course of the underlying disease, (iii) laboratory data, (iv) antibody response to SARS-CoV-2, and (v) IT modifications, where needed.

## 2. Methods and Patients

### 2.1. Study Design

This is an observational single center case series of four consecutive patients with AID, who were diagnosed or highly suspected for COVID-19 in March 2020. All IL-1-mediated AID patients were treated at the time of COVID-19 symptom onset with IT according to international recommendations [3,4]. The patients had a follow-up of 100 days. At the follow-up visits, therapy was adjusted according to established treat-to-target strategies if necessary [29]. The patients’ data were captured in a standardized way in the designated institutional web-based Arthritis and Rheumatism Database and Information System (ARDIS) [30]. Individual patient’s informed consent was obtained for data analysis and publication. A waiver of ethical approval was obtained from the University of Tuebingen Institutional Review Board (951/2020A).

### 2.2. Patients

#### 2.2.1. Patient 1

Patient 1 is a 34-year-old woman diagnosed with an unclassified AID with undifferentiated arthritis. Disease symptoms within the last 12 years were recurrent fever of 1–2 days duration every 3 to 10 weeks including highly elevated inflammatory parameters during flares, abdominal pain with diarrhea, oral aphthosis, and arthritis, mainly affecting the palmar joints. A comprehensive work-up including clinical and laboratory examinations and imaging excluded malignancies, immunodeficiencies, autoimmune diseases and infections. The institutional genetic AID panel test for common AID (e.g., AID caused by (i) IL-1, (ii) interferon, (iii) nuclear factor kappa B (NF-kB) dysregulation in keratocytes, (iv) NF-kB also affecting interferon signaling, (v) NF-kB dysregulation and granulomatous diseases and (vi) systemic macrophage activation) was negative. Treatment for arthritis was started with sulfasalazine but without any improvement. Therefore, treatment was changed to adalimumab and later to etanercept. Both biological disease modifying antirheumatic drugs (bDMARDs) had to be stopped due to side effects. For the last nine years, she has been treated with subcutaneous methotrexate (MTX) with dose increases during recent years, finally up to 20 mg weekly, resulting in mild disease activity (physician global assessment (PGA) 2).

#### 2.2.2. Patient 2

Patient 2 is a 14-year-old girl. She lives in the same household as patient 1, who is her mother, and was diagnosed at the age of 9 years with CAPS. Since her late infancy she experienced recurrent episodes of fever, musculoskeletal complaints, gastrointestinal symptoms and fatigue coupled with severely elevated C-reactive protein (CRP) and serum amyloid A (SAA) during disease flares. A comprehensive work-up excluded malignancies, immunodeficiencies, autoimmune diseases and infections. The institutional genetic AID test detected a heterozygous p.Q703K variant in the *NLRP3* gene. As this low-penetrance variant can be associated with fevers and gastrointestinal symptoms [31], the variant was postulated as being causative for her disease symptoms. Anakinra 2 mg/kg/day was started due to moderate to high disease activity, as partial to good response to anti-IL-1 treatment has been reported for these variants [31]. At the age of 11 years a switch to canakinumab 150 mg/every 4 weeks (q4w) was established, resulting in no to mild disease activity during the last three years (PGA ≤ 2).

#### 2.2.3. Patient 3

Patient 3 is the 12-year-old brother of patient 2 and the son of patient 1. He was diagnosed with CAPS (heterozygous *NLRP3* p.Q703K variant) at the age of 7 years after exclusion of malignancies, immunodeficiencies, autoimmune diseases and infections. Since age 5, he had recurrent fevers with urticaria-like rashes, abdominal pain, arthralgia/arthritis and highly elevated inflammatory markers (SAA and CRP) during flares. Treatment was started immediately, after CAPS diagnosis, with anakinra 2 mg/kg/day resulting in prompt improvement of high disease activity. A switch to canakinumab injections at 4 mg/kg/q4w was performed for better compliance. During recent years he achieved a stable mild to moderate disease activity on canakinumab 150 mg s.c/q4w (PGA 1–3).

#### 2.2.4. Patient 4

Patient 4 is a 15-year-old girl diagnosed with FMF (homozygous p.M694V variant in the *MEFV* gene) at four years of age. Treatment was started with colchicine 0.5 mg/day with a stepwise dose increase due to persistent high clinical and laboratory disease activity. As the patient showed colchicine resistance at 1.5 mg/day and intolerance at 2 mg/day colchicine, anti-IL-1 treatment was started at the age of six years according to recommendations for FMF [3]. Treatment response was achieved with anakinra 2 mg/kg/day combined with colchicine 1 mg/day. Daily injections were not well tolerated and therefore therapy was switched to canakinumab 2 mg/kg/q4w and later 150 mg/q4w. During the last three years she showed no to mild disease activity (PGA ≤ 2).

### 2.3. Monitoring and Follow-Up Visits

The patients’ monitoring included physical examination, laboratory assessments and symptom diaries. The patients captured their daily AID symptoms in a diary similar to the Autoinflammatory Disease Activity Index (AIDAI) [32]. Disease activity was assessed for the underlying AID by the physician at each clinical visit. Disease activity was defined as physician global assessment (PGA), recorded on a 10 cm visual analog scale (VAS) with 0 representing no disease activity and 10 maximum disease activity. Furthermore, disease activity was assessed by the patients (PPGA) and recorded on a 10 cm VAS comparable to the PGA. Laboratory monitoring included the inflammatory markers CRP, SAA, S-100 proteins, and erythrocyte sedimentation rate (ESR). Additionally, blood count, liver enzymes and kidney function tests were performed. SARS-CoV-2 antibody-tests were performed for each patient during follow-up visits, if the patient and their parents agreed to the test. Antibodies were detected by ELISA with an in-house assay as published previously [33]. Briefly, microtiter plates were coated with the SARS-CoV-2 proteins nucleocapsid, spike 1 (both obtained from SinoBiological; Peking, China), and the RBD-spike 1 protein (GenScript; New Jersey, USA) at concentrations of 0.1 µg/mL, 0.2 µg/mL, and 0.3 µg/mL, respectively. Patients’ sera were used at a dilution of 1:500 for the demonstration of IgG- and IgM-antibodies and 1:100 for the detection of IgA-antibodies. Bound antibodies were detected with peroxidase conjugated goat anti-human IgG-, IgM- and IgA-antibodies (DIANOVA, Hamburg, Germany) at dilutions of 1:3000, 1:2000, and 1:650, respectively. As substrate o-phenylendiamine was used. Reactivity was given as arbitrary units (AU). Optimal antigen- and serum dilutions have been evaluated by serial dilutions prior to analysis. In each assay four sera with defined AU (high, medium, low, and negative) were tested as standard sera. Applying all three antigens in parallel, sensitivity of the assay for the demonstration of anti-SARS-CoV-2-antibodies was 97% and specificity 99%.

## 3. Results

### 3.1. Acute COVID-19 Course

#### 3.1.1. Patient 1

**Figure 2:**
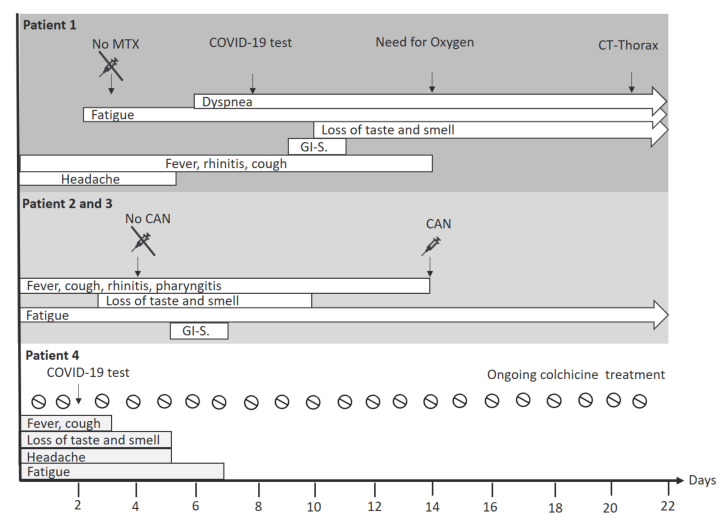
Overview of symptoms of the acute COVID-19 disease course in AID patients. Acute COVID-19 disease course was less severe in the three adolescent AID patients with canakinumab maintenance treatment as compared to the adult patient treated with methotrexate. Particularly patient 4, who was treated with daily colchicine and who had received canakinumab 150 mg s.c. 4 days before COVID-19 was confirmed, had a short and mild disease course. Abbreviations: CAN: Canakinumab; CT: Computed tomography; COVID-19: Coronavirus disease 2019; GI-S.: Gastrointestinal symptoms such as nausea, diarrhea, emesis, abdominal pain; MTX: Methotrexate. Legend: ⇨ ongoing disease symptoms on day 22 after COVID-19 onset.

At day one of disease onset, patient 1 developed rhinitis, fever, headache, fatigue and cough (Figure 2). On day 10, she reported a loss of taste and additionally she complained about nausea, emesis and abdominal pain. On day 14, home oxygen was started due to respiratory insufficiency. On day 21, a computed tomography of the lungs was performed showing typical signs of ground-glass opacities. MTX was administered four days before symptom onset and was discontinued when first symptoms suggestive for COVID-19 appeared. During the acute episode, no laboratory work-up was performed.

#### 3.1.2. Patient 2 and 3

Disease courses in patients 2 and 3 were similar. They developed fever, cough, pharyngitis, fatigue and rhinitis ten days after first disease symptoms of patient 1 (Figure 2). Between day 3 and 13, both reported a loss of taste and smell. On day six, they complained about nausea and abdominal pain. Patient 2 additionally had diarrhea. At day 14 both siblings recovered and only persistent fatigue was reported. Canakinumab was administered at day 14 after symptom onset, with a total delay of 10 days compared to usual dosing regimen (Figure 2). No laboratory work-up was performed during the acute disease course.

#### 3.1.3. Patient 4

Patient 4 developed fever, cough, loss of taste and smell, and headache four days after her last canakinumab injection (Figure 2). Cessation of cough and fever was reported two to three days after symptom onset. Disappearance of headache and normalized smell and taste was reported 5 days after the onset of COVID-19 symptoms. Fatigue was present until day 7. Recovery was reported since day 8. Canakinumab injections and also colchicine treatment were continued (Figure 2).

### 3.2. Follow-Up Visits

#### 3.2.1. Patient 1

At first follow-up visit patient 1 still suffered from severe fatigue, loss of taste and smell, dyspnea and ongoing muscoloskeletal complaints with arthralgia (Table 1). PGA was estimated with 2. The ESR was mildly elevated but otherwise the laboratory results were normal (Table 2). MTX was restarted at day 90. At second follow-up taste and smell were normal, but fatigue and dyspnea during physical exercises were still present. Inflammatory parameters were no longer elevated and disease activity was stable (Table 1 and Table 2).

#### 3.2.2. Patient 2

Patient 2 reported ongoing mild to moderate fatigue and intermittent mild erythematous macular rash at the first follow-up visit. The PGA showed mild disease activity, whereas the patient estimated disease activity as moderate (Table 1). No ongoing inflammation was detected (Table 2). At second follow-up mild rash and fatigue persisted. One mild flare was reported. No inflammation was detected (Table 1 and Table 2).

#### 3.2.3. Patient 3

At the first follow-up patient 3 reported moderate erythematous macular rash, severe fatigue and arthralgia (Table 1). PGA and PPGA had increased to 4 and 7, respectively. However, laboratory results revealed no inflammation (Table 2). Between the first and second follow-up he showed typical signs of active CAPS with persistent urticarial-like rashes, conjunctivitis, sever fatigue, intermittent subfebrile temperatures and arthralgia. Additionally he complained about mild abdominal pain without signs of peritonitis, diarrhea or nausea. Vital signs, liver enzymes, kidney function tests, creatinine kinase, fibrinogen, ferritin, thrombocytes and hemoglobin were normal. The canakinumab dose was increased to 300 mg/q4w. At the second follow-up, symptoms were still present but milder (Table 1), and inflammatory markers could not be detected anymore (Table 2).

#### 3.2.4. Patient 4

Patient 4 reported neither COVID-19 nor FMF symptoms at the first and second follow-up visits (Table 1). Laboratory parameters did not change during the follow-up period (Table 2).

### 3.3. Nasopharyngeal COVID-19 Tests and Antibody Response to SARS-CoV-2

Patient 1 was tested with nasopharyngeal COVID-19 test (RT-PCR) on day 8 after onset of COVID-19 symptoms and received a positive test result on day 11. Patient 2 and 3 were tested negative two days before their first COVID-19 symptoms occurred. After symptom onset, both were diagnosed clinically for COVID-19 due to suggestive symptoms and close contact to a person with confirmed COVID-19. Patient 4 was tested positive two days after onset of COVID-19 symptoms. At first follow-up visit, the antibody response to SARS-CoV-2 was tested in patient 2, confirming that she had COVID-19. At day 100 COVID-19 post-infection three out of four patients (75%) showed no antibody response to SARS-CoV-2 (Table 3).

## 4. Discussion

This is one of the first case series illustrating the acute COVID-19 disease course and follow-up data in patients with IL-1-mediated and unclassified AID treated with IT. All patients had common COVID-19 symptoms and no one needed admission to the intensive care unit. The acute COVID-19 disease course was milder in the adolescent patients treated with maintenance anti-IL-1 treatment compared to the adult patient with maintenance MTX. Although all patients recovered, fatigue was a common long-lasting symptom reported by 75% of patients after acute COVID-19. One patient with CAPS experienced increased AID disease activity necessitating adjustment of anti-IL-1 treatment (canakinumab 300 mg/q4w) a few weeks after acute COVID-19 disease course. At day 100 post COVID-19-infection, in three out of four patients (75%), SARS-CoV-2 antibodies were undetectable.

### 4.1. COVID-19 Disease Course and AID

AID patients with IT and controlled AID activity can show typical COVID-19 symptoms, but seem not to be at increased risk for severe acute COVID-19. Typical COVID-19 symptoms are fever, respiratory symptoms (rhinitis, dyspnea, and coughing), sore throat, loss of taste/smell, muscle pain additionally headache, and gastrointestinal symptoms [34,35,36,37,38]. The patients in this case series showed common COVID-19 symptoms. All four patients had IT, but none had a severe disease course. These observations are in line with previous published data showing that patients with IT, particularly children, seem not to be at increased risk to develop (i) COVID-19 or (ii) a more severe disease course or (iii) an inferior outcome after being infected with COVID-19 in comparison with the general population [26,27,28,39,40,41].

Data for patients with AID are scarce. Haslak et al. studied 404 AID patients (90% FMF, 3.4% CAPS, 0.2% TRAPS, 6.1% others) with COVID-19 [25]. He reported six FMF patients with colchicine tested positive for COVID-19 and all recovered completely [25]. Additionally, he reported one asymptomatic CAPS patient with canakinumab, who was tested negative for COVID-19 although he had close contact to family members with confirmed COVID-19 [25]. Similarly, Koker et al. reported negative test results for an asymptomatic FMF patient and a CAPS patient suffering from arthralgia, both with maintenance anti-IL-1 treatment and close contact to family members with confirmed COVID-19 [27]. A 70-year-old CAPS patient treated with canakinumab 150 mg/q8w and confirmed COVID-19 showed a very mild clinical disease course with a soon recovery [42]. Usually, a 70-year-old patient would be regarded at high risk for experiencing a serious COVID-19 disease course. Therefore, the authors hypothesized that cytokine blockade may protect from cytokine storm and thus ameliorate the gravity of the clinical picture of COVID-19 [42]. COVID-19 seems to be associated with a massive inflammatory response that appears to occur via stimulation of the NLRP3 inflammasome [43]. The NLRP3 inflammasome plays also an important role in mediating systemic inflammation in CAPS (Figure 1). This raises the questions if (i) patients with NLRP3 inflammasome-associated cytokine release are at higher risk of hypercytokinemia and increased AID activity during or after acute COVID-19; and if (ii) maintenance anti-IL-1 treatment, applied in IL-1-mediated AID to avoid inflammasome activation and cytokine release, also reduces COVID-19-related cytokine dysregulation accounting for mild or asymptomatic COVID-19 disease courses in these patients. The three adolescent patients with IL-1-mediated AID and anti-IL-1 treatment in this case series had very mild COVID-19 symptoms. Whereas the FMF patient did not show any signs of increased AID activity, patient 2 reported mildly increased disease activity without need for therapy adjustment during follow-up. Patient 3 required dose increase of anti-IL-treatment (canakinumab 300 mg/q4w) a few weeks after acute COVID-19, due to moderate to severe AID activity compared to pre-COVID-19 era. Although canakinumab was administered with a delay of 10 days in patient 2 and 3 it can be expected that the IL-1β blocking effects were still present. Canakinumab has a long half-live (t_1/2_) of 28 days, so that from a pharmacologic point of view 95–99% of the drug will be eliminated after 84–140 days. In comparison to the adolescent patients, patient 1 had a more severe COVID-19 disease course, which might have several reasons (e.g., age, other risk factors). In patient 1 maintenance MTX treatment was stopped with symptom occurrence. Despite the relatively short MTX plasma half-life, MTX can persist intracellularly in red blood cells as MTX polyglutamate (MTXGlu). The median half-life elimination of MTXGlu ranges from 1.2 to 4.3 weeks, resulting in a median time of 15 weeks after MTX cessation (range 3 to ≥32 weeks) to become undetectable [44]. This case series indicates that patients with IL-1-mediated or unclassified AID with maintenance IT can experience typical COVID-19 symptoms without AID flares during acute COVID-19 and seem not to be at an increased risk for a severe acute COVID-19 disease course. However, follow-up visits are important as similarly to other infections COVID-19 can increase the underlying AID activity a few weeks later with the need for therapy adjustment.

### 4.2. Antibody Response to SARS-CoV-2

In three out of four patients in this case series, no SARS-CoV-2 antibody response was detectable at day 100. There is evidence that most patients seroconvert to SARS-CoV-2 specific IgG antibodies within 2 to 4 weeks after symptom onset [45,46,47]. Murchu et al. reviewed 74 studies and reported, that IgG could be detected in all reviewed patients (*N* = 24) at 49–65 days and that neutralizing antibodies were detected in 92%–100% of patients up to 53 days [45]. SARS-CoV-2 antibody decline after COVID-19 is under current research. Decrease of COVID-19 immunity might be possible similar as seen in other coronaviridae [45,48]. Seow at al. detected high neutralizing antibody titers at >60 days in individuals with high peak infective doses, whereas patients with lower peaks returned to baseline over a relatively short period suggesting decrease of COVID-19 immunity [49]. To differentiate between re-infection and persistent viral shedding, To et al. performed a comparative genome virus analysis in a patient with a second episode of COVID-19 symptoms 142 days after the first confirmed COVID-19 episode and found a re-infection by a different strain [50]. Similarly, Tillett et al. detected genetically significant differences between virus variants in a patient tested positive in April and June for COVID-19 [51]. Whereas Tillett et al. reported that the second episode was symptomatically more severe than the first, Bentivegna et al. reported an asymptomatic female patient at her second COVID-19 episode [51,52]. Interestingly, she was tested positive for SARS-CoV-2-specific IgG (CLIA assay) after onset of COVID-19 symptoms [52]. Torres et al. reported an otherwise healthy female patient with COVID-19 reinfection 12 weeks after first confirmed COVID-19 [53]. At the first COVID-19 episode she was tested negative for IgG antibody response to SARS-CoV-2 at day 23, 33 and 67 [53]. Although Freeman at al. discovered that pediatric immunocompromised patients are capable of producing an antibody response to SARS-CoV-2, they reported that one out of two documented RT-PCR positive patients did not show any seroconversion [54].

At day 100, three out of four patients (75%) in our case series had no detectable antibody response. As one of these patients displayed SARS-CoV-2 antibodies at day 40, antibody loss can be postulated for this patient. For the two others patients, it cannot be determined if they (i) did not develop antibodies or (ii) had a loss of immunity. We were unable to find (i) any other follow-up data on antibody response to SARS-CoV-2 in patients with IT, immunosuppressive diseases or AID; (ii) data on COVID-19 reinfection in this particular patient group; or (iii) long-term follow-up data for antibody-responses in immunocompetent patients. Taken together, we can summarize that SARS-CoV-2 antibody decline is currently incompletely understood. The sparse data from this case series might raise the question of whether patients with IT might have a risk for no seroconversion or loss of antibody response to SARS-CoV-2. More research in this area is necessary and antibody monitoring in patients with IT might help to better understand their risk regarding COVID-19 reinfection. It is important to be aware of the possibility of COVID-19 re-infection in patients with IT.

### 4.3. Limitations

This case series has several limitations. First of all, the sample size is small. However, AID are orphan diseases and therefore, data of COVID-19 confirmed AID patients is rare. To our knowledge this is the first case series reporting follow-up data of COVID-19 in IL-1-mediated and undifferentiated AID patients. Moreover, particularly regarding laboratory values, there is missing data. All patients were diagnosed at the end of March through the first wave of the COVID-19 pandemic and the start of lockdown in several countries in Europe. March was a very challenging month for the health care systems in Europe as COVID-19 test resources were limited, SARS-CoV-2 antibody tests initially were not available and several university hospitals could not schedule regular visits for their chronically ill/immunosuppressed outpatients. Consequently, some diagnostic tests—now being well established—were not done regularly. In addition, some missing data can be explained as we report patients from a real-life cohort. However, standardized outcome evaluation of the included patients combined with advanced laboratory testing resulted in comparable high-quality data captured in ARDIS. Antibody response was only tested using ELISA for all patients at day 100 and only in patient 2 at day 40. Additional nasopharyngeal COVID-19 RT-PCR was not performed at day 40 and 100, due to the reasons explained above. Although data from this case series are not generalizable, due to small sample size, important insights can be gained which may be taken into account in case AID patients with and without IT develop COVID-19.

## 5. Conclusions

Patients with IL-1-mediated or unclassified AID and maintenance IT can experience typical COVID-19 symptoms, but seem not to be at an increased risk for severe acute COVID-19 disease course. However, follow-up visits are important to monitor AID activity as COVID-19 may increase underlying AID activity a few weeks after acute infection, necessitating IT dose adjustments. In this case series, only one patient had detectable antibodies against SARS-CoV-2 at day 100. Therefore, AID patients with IT should be monitored carefully for new COVID-19 symptoms and should be re-tested if indicated. Data from international registries and follow-up data combination of COVID-19 in AID and monitoring of SARS-CoV-2 antibodies will help to better understand COVID-19 and SARS-CoV-2 immunity in AID and the role of IT.

## Figures and Tables

**Table 1:** Clinical symptoms before and after COVID-19 symptom onset.

	PGA (0–10 cm)	PPGA (0–10 cm)	Flares/3 Months	Fever	Rash	Fatigue	Dyspnea	Arthralgia
**Patient 1**								
Before COVID-19	2	2	1	-	-	+	-	+
~Day 40 *	2	n.a.	n.a.	-	-	+++	++	+
~Day 100 *	2	n.a	n.a.	-	-	++	+	+
**Patient 2**								
Before COVID-19	≤2	≤2	0	-	-	-	-	-
Day 40 *	2	5	n.a	-	+	+/++	-	-
Day 100 *	2	2	1	-	+	+	-	-
**Patient 3**								
Before COVID-19	1–3	2	0	-	-	+	-	-
Day 40 *	4	7	n.a.	-	++	+++	-	+
Day 100 *^,1^	5	7	3	+/++	-	+++	-	++
**Patient 4**								
Before COVID-19	≤2	0	0	-	-	-	-	-
Day 40 *	2	n.a.	n.a.	-	-	-	-	+
Day 100 *	1	0	0	-	-	-	-	-

Abbreviations: PGA: physician global assessment recorded on a visual analog scale with 0= no disease activity and 10= maximum disease activity; PPGA: Disease activity estimated by the patient similar recorded to the PGA; n.a.: not available, Symptom severity: - absence, + mild, ++ moderate, +++ severe; * after COVID-19 onset; ^1^ five days after Canakinumab increase (300 mg/q4w).

**Table 2:** Laboratory results before and after COVID-19 symptom onset.

	Hb (g/dl)	Leuc (10S9/l)	Plt (10S9/l)	ESR (mm/h)	CRP (mg/dl)	IL-6 (ng/L)	S100A8/A9 (μg/mL)	sl-ILR2 (U/mL)	SAA (mg/L)
**Patient 1**									
Before COVID-19	11.8	5.52	197	8	0.34	<2.0	2.9	n.a.	2
~Day 40 *	12.4	5.30	256	22	0.72	3.0	16.4	267	4
~Day 100 *	11.6	5.92	231	13	1.54	n.a.	6.2	n.a.	6
**Patient 2**									
Before COVID-19	13.5	4.30	303	5	0.01	<2.0	11.3	n.a.	1
Day 40 *	14.3	5.09	239	n.a.	0.01	2.7	10.4	215	2
Day 100 *	13.9	5.38	301	2	0.01	n.a.	6.3	n.a.	1
**Patient 3**									
Before COVID-19	14.3	5.37	319	2	0.01	<2.0	3.2	n.a.	1
Day 40 *	15.0	5.63	305	4	0.02	2.7	6.9	164	2
Day 100 *^,1^	14.5	5.78	300	2	0.01	2.8	5.7	n.a.	2
**Patient 4**									
Before COVID-19	13.2	5.54	191	13	0.14	2.9	64.4	n.a.	19
Day 40 *	13.3	4.61	180	2	0.05	n.a.	n.a.	n.a.	10
Day 100 *	12.5	4.06	174	7	0.07	3.7	40.9	n.a	15

Abbreviations: Hb: Hemoglobin; Leuc: Leucocytes; Plt: Platelets; ESR: Erythrocyte sedimentation rate; CRP: C-reactive protein, IL-6: Interleukin-6; S100A8/A9: S100 protein A8/A9; sl-ILR2: soluble Interleukin Receptor 2; SAA: Serum Amyloid A; n.a.: not available; * after COVID-19 symptom onset; ^1^ five days after increased Canakinumab dose (300 mg/q4w).

**Table 3:** Test results for COVID-19/SARS-CoV-2 antibodies.

	COVID-19 Test (Nasopharyngeal)	SARS-CoV-2 Antibodies ^§^ (Serum)
**Patient 1**		
Day 8 *	Positive	n.a.
~Day 40 *	n.a.	n.a.
~Day 100 *	n.a.	Negative
**Patient 2**		
Day -2 (before COVID-19 symptom onset)	Negative	
Day 40 *	n.a.	Positive IgG (19.4 U/mL) and IgA (15.2 U/mL) SARS-CoV-2-nucleocapside, Positive IgG SARS-CoV-2-spike (18.3 U/mL), Positive IgG SARS-CoV-2-RBD (19.9 U/mL)
Day 100 *	n.a.	Negative
**Patient 3**		
Day -2 (before COVID-19 symptom onset)	Negative	n.a
Day 40 *	n.a.	n.a.
Day 100*^,1^	n.a.	Positive IgG (59.3 U/mL) and IgA (28.6 U/mL) SARS-CoV-2-nucleocapside
**Patient 4**		
Day 2*	Positive	n.a.
Day 40*	n.a.	n.a.
Day 100*	n.a.	Negative

Abbreviations: n.a.: not available; * after COVID-19 symptom onset; ^1^ five days after canakinumab increase (300 mg/q4w), ^§^ Antibody against SARS-CoV-2-spike, SARS-CoV-2-RBD, SARS-CoV-2-nucleocapside, Reference values: SARS-CoV-2-nucleocapside: IgG >11.0 U/mL, IgA >4.4 U/mL; SARS-CoV-2-spike: IgG >15.0 U/mL, IgA >6.0 U/mL, SARS-CoV-2-spike-RBD: IgG >15.0 U/mL, IgA >14.0 U/mL.

## Data Availability

Data is contained within the article.
